# Physiological and biochemical responses involved in water deficit tolerance of nitrogen-fixing *Vicia faba*

**DOI:** 10.1371/journal.pone.0190284

**Published:** 2017-12-27

**Authors:** Ablaa Kabbadj, Bouchra Makoudi, Mohammed Mouradi, Nicolas Pauly, Pierre Frendo, Cherki Ghoulam

**Affiliations:** 1 Unit of Plant Biotechnology and Symbiosis Agrophysiology, Faculty of Sciences and Techniques, Guéliz, Marrakesh, Morocco; 2 Université Côte d'Azur, INRA, CNRS, ISA, France; Institute for Sustainable Plant Protection, C.N.R., ITALY

## Abstract

Climate change is increasingly impacting the water deficit over the world. Because of drought and the high pressure of the rising human population, water is becoming a scarce and expensive commodity, especially in developing countries. The identification of crops presenting a higher acclimation to drought stress is thus an important objective in agriculture. The present investigation aimed to assess the adaptation of three *Vicia faba* genotypes, Aguadulce (AD), Luz d’Otonio (LO) and Reina Mora (RM) to water deficit. Multiple physiological and biochemical parameters were used to analyse the response of the three genotypes to two soil water contents (80% and 40% of field capacity). A significant lower decrease in shoot, root and nodule dry weight was observed for AD compared to LO and RM. The better growth performance of AD was correlated to higher carbon and nitrogen content than in LO and RM under water deficit. Leaf parameters such as relative water content, mass area, efficiency of photosystem II and chlorophyll and carotenoid content were significantly less affected in AD than in LO and RM. Significantly higher accumulation of proline was correlated to the higher performance of AD compared to LO and RM. Additionally, the better growth of AD genotype was related to an important mobilisation of antioxidant enzyme activities such as ascorbate peroxidase and catalase. Taken together, these results allow us to suggest that AD is a water deficit tolerant genotype compared to LO and RM. Our multiple physiological and biochemical analyses show that nitrogen content, leaf proline accumulation, reduced leaf hydrogen peroxide accumulation and leaf antioxidant enzymatic activities (ascorbate peroxidase, guaiacol peroxidase, catalase and polyphenol oxidase) are potential biological markers useful to screen for water deficit resistant *Vicia faba* genotypes.

## Introduction

Due to global warming and the rising human population, water availability is becoming an increasing issue for agriculture. One of the main challenges of modern agriculture is to maintain growth and crop productivity under adverse environmental stress conditions such as water deficit. Water deficit impairs plant development leading to consequent loss of crop yield. It has been reported that more than 50% of the average yield of most major crops is lost due to drought stress [1]. Under the on-going climate change scenario in the world, increase of drought severity and frequency has been predicted to further increase in the near future [2]. Given that water deficit constitutes a crucial constraint to plant growth and development, plants have developed physiological, cellular and molecular responses leading to drought stress adaptation and consequently to better plant growth under stress conditions.

When plants grow in a water-limited environment, they undergo physiological, and biochemical modifications considered as water deficiency adaptation mechanisms [3]. Growth inhibition is often associated with altered plant water status, with a decrease in the relative water content in leaves [4,5]. At the physiological level, the ability of plants to maintain the functioning of the photosynthetic machinery under water stress is of major importance in drought tolerance [6]. Besides alterations to plant metabolism, drought stress progressively decreases CO_2_ assimilation rates due to reduced stomatal conductance [7]. Total chlorophyll and carotenoid contents were also found to decline under water restricted conditions [8,7]. Also, alteration of plant metabolism during environmental stresses such as water deficit, induces the accumulation of reactive oxygen species (ROS) in the cells, causing oxidative damage to the plants [9,10].

Plants have evolved many mechanisms for drought stress tolerance including a number of physiological and biochemical processes such as maintenance of water-use efficiency, osmotic adjustment and protection of the cellular machinery [11,12,13]. Osmotic adjustment is involved in plant resistance to lowered soil water potential, which decreases water availability. In many plants, osmotically active solutes allow a partial maintenance of turgor dependent processes under water stress conditions [14]. Plants also increase antioxidant defence through synthesis of antioxidants such as ascorbate and glutathione, and enhance antioxidant enzymes such as peroxidase, superoxide dismutase and catalase to scavenge the ROS produced during stress [12,15,16].

Faba bean (*Vicia faba* L.) is considered as one of the most extensively grown grain legumes cultivated in diverse environments [17]. It has high grain protein content, ranging from 20 to 41% [18]. Besides its use as food and feed, faba bean can establish a nitrogen-fixing symbiosis with soil bacteria of the rhizobium family, representing a major source of nitrogen (N) input in agricultural soils when faba bean is included in crop rotation [19,20]. The production of this crop is increasingly challenged by the growing population food demand and drought is a major abiotic constraint responsible for heavy faba bean production losses [21]. Faba bean is more sensitive to drought than some other grain legumes including common bean, pea and chickpea [22,3]. In this context, development of faba bean genotypes resistant to water limited conditions through breeding is an important strategy for improving food security of small farmers in North Africa. The response of faba bean to drought stress has been analysed to identify physiological processes and biological markers associated with drought tolerance [23,24,25]. Direct measurement of physiological processes involved in drought response is a useful and pragmatic option for screening multiple genotypes [26]. Considering the importance of faba bean for humans and animals, the present work has been performed to study the effect of water deficit on different genotypes cultivated in Morocco. We performed a broad analysis by measuring numerous physiological and biochemical parameters on three faba bean cultivars. The main objectives of the present investigation were to define whether these three common Moroccan cultivars presented differences in their ability to tolerate water deficit, then to explore the relation between resistance to water deficit and plant physiological and biochemical markers. The analysis of our results led to the identification of potentially useful traits that may be used in breeding programs for production of faba bean genotypes tolerant to water deficit stress or identify easy to use biological markers, which can be used as indirect selection criteria for genotype drought tolerance characterisation.

## Materials and methods

### Biological material and growth conditions

Three faba bean genotypes, Aguadulce (AD), Luz d’Otono (LO) and Reina Mora (RM), frequently grown at the Haouz region of Morocco, were used in this study. Before sowing, seeds of all genotypes were surface sterilized with 2.5% sodium hypochlorite for 20 min, then were washed with sterile deionized water and left to germination in sterile sand at 23°C for five days. Seedlings with homogenous stages of development were selected and put in pots filled with a mixture of sterilised sand and peat (4:1). Then, they were inoculated with a *Rhizobium leguminosarum* (strain Fb41) suspension at 10^8^ bacteria per mL. The experiments were conducted in a greenhouse (approximate temperature of 26/20°C (day/night), 50–80% of relative humidity and 16 h photoperiod) at the Faculty of Sciences and Techniques, Marrakesh (31° 38' 2 N, -7° 59' 59 W, 1529 ft). Water deficit was initiated when seedlings were almost 20 day-old, attaining 2–3 true leaves and it was applied by maintaining the soil moisture at 40% of substrate field capacity (FC) for the stressed plants, and 80% FC for the well-watered plants. The experimental plant pots were arranged in a simple randomized design with twelve replicates per treatment per genotype. The nutrient solution applied once a week was composed as follows: MgSO_4_ (100 μmol.L^-1^), K_2_SO_4_ (750 μmol.L^-1^), KH_2_PO_4_ (125 μmol.L^-1^), CaCl_2_ (1650 μmol.L^-1^), Sequestrene (16 μmol.L^-1^), MnSO_4_ (6 μmol.L^-1^), H_3_BO_3_ (4 μmol.L^-1^), ZnSO_4_ (1 μmol.L^-1^), NaMoO_4_ (0.1 μmol.L^-1^), and CuSO_4_ (1 μmol.L^-1^). At plant flowering stage, about 50–60 day-old plants were harvested for growth assessment, and physiological and biochemical analyses.

### Dry weight and carbon/nitrogen balance measurements

Shoot dry weight (SDW), root dry weight (RDW) and nodule dry weight (NDW) were measured for the three faba bean genotypes. Once harvested, shoots, roots and detached nodules were washed and dried at 70°C for 48 h. Three replicates (six plants for each treatment per genotype) were performed. For nitrogen (N) and carbon (C) analyses, samples (root, shoot, nodules) from six plants were considered as three replicates and were analysed using a CHN Elemental Analyser (Fison EA 1108; Thermo Scientific Waltham, USA).

### Relative water content and leaf mass per area

The relative water content (RWC) was determined on leaf discs of 1 cm diameter from fully expanded third leaves and calculated as:

$$RWC\;(\%)=\left(\frac{FW-DW}{TW-DW}\right)*100$$ (1)

Where FW is the fresh weight, TW is the turgid weight after water saturation of the discs at 4°C and DW is the dry weight after samples drying at 75°C until obtaining a constant weight [27]. The leaf mass per area (LMA) was determined as DW/leaf area. Leaf area was measured beforehand using image analysis with Image J software (http://rsb.info.nih.gov/ij/index.html). Three replicates (six plants for each treatment per genotype) were performed.

### Chlorophyll fluorescence

Maximum quantum efficiency of Photosystem II (PSII) (Fv/Fm) in individual mature leaves was analysed using a fluorescence meter (Handy PEA, Hansatech, England) according to the method of Jifon and Syvertsen [28]. After 35 days of water stress, the fully expanded third leaves were dark-adapted for 30 min before measurement to therefore determine the Minimal fluorescence yield (Fo) and Maximal fluorescence yield (Fm). Subsequently, the Fv/Fm [Fv/Fm = (Fm—Fo)/Fm] was obtained automatically by the device. Three replicates per treatment were performed.

### Photosynthetic pigment content

Photosynthetic pigments were measured as described by Takele [29]. Chlorophyll and carotenoid contents were determined from 0.2 g leaf tissues. After 35 days of water stress, leaf segments from fully expanded third leaves were cut into small pieces and extracted in 4 mL of acetone (80%, V/V) for 48 h at 4°C. The absorbance of the extracts was measured at 661.6, 644.8, and 470 nm. Chlorophyll (a + b) and carotenoid (x + c) contents were calculated as described by Lichtenthaler [30] and expressed as mg mL^-1^ g^-1^ DW. Total chlorophyll was determined using the following formula:

$$C_{a+b}=7.15*A_{663.2}+18.71*A_{646.8}$$ (2)

$$C_{x+c}=\frac{1000*A_{470}-1.82*C_{a}-85.02*C_{b}}{198}$$ (3)

Four replicates per treatment were performed.

### Osmoprotectant contents

After 35 days of water stress, plants were harvested and samples from fully expanded third leaves, roots and nodules were collected for osmoprotectant content measurements. Free proline was extracted from 0.2 g of fresh plant material samples in aqueous sulphosalicylic acid (3%, W/V) and estimated by using ninhydrin reagent according to the method of Bates et al. [31]. The absorbance of fraction with toluene aspired from liquid phase was read at 520 nm. Proline concentration was determined using calibration curves and expressed as nmol proline g^-1^ FW. Three replicates per treatment were performed.

Glycine betaine (GB) was determined by the method described previously by Grieve and Grattan [32] with some modifications. Dried plant material (0.25 g) was finely powdered and shaken with 10 mL of deionized water for 48 h at 25°C. The extracts were diluted 1:1 with 2N H_2_SO_4_. Five hundred microliter were collected and cooled in ice for 1 h, before adding 200 μL of cold KI-I_2_ reagent. The tubes were stored at 4°C for 16 h and then centrifuged at 10 000 rpm for 15 min at 4°C. The supernatant was removed. Periodite crystals were dissolved in 4 mL of 1,2-dichloroethane. After 2 h, the absorbance was measured at 365 nm. GB concentration was determined using calibration curves and expressed as nmol glycine betaine g^-1^ DW. Three replicates per treatment per genotype were performed.

### Lipid peroxidation and hydrogen peroxide content

After 35 days of water stress, plants were harvested and samples from fully expanded third leaves, roots and nodules were collected for oxidative stress indicator measurements. Lipid peroxidation was determined by estimating the malonyldialdehyde (MDA) content in 0.2 g leaf fresh weight according to Madhava Rao and Sresty [33]. MDA is a product of lipid peroxidation by thiobarbituric acid reaction. The MDA concentration was calculated from the absorbance at 532 nm (unspecific turbidity was corrected by subtracting the absorbance at 600 nm) by using an extinction coefficient of 155 mM^-1^ cm^-1^. Three replicates per treatment per genotype were performed.

Hydrogen peroxide (H_2_O_2_) was measured as described by Velikova *et al*. [34]. Fresh plant material samples (0.2 g) were homogenized in 4 mL of 20% (w/v) tricarboxylic acid. The homogenate was centrifuged at 12 000 rpm for 15 min at 4°C. The supernatant was then added to 10 mM potassium phosphate buffer (pH 7.0) and 1 M potassium iodide. The absorbance of the supernatant was measured at 390 nm. The H_2_O_2_ content was calculated by comparison with a standard calibration curve plotted using known concentrations of H_2_O_2_. Each treatment included three replicates.

### Determination of antioxidant enzymes activities

After 35 days of water stress, crude enzyme extracts made from fully expanded third leaves, roots and nodules were used to determine the activities of antioxidant enzymes. Fresh plant tissue from 0.2 g was homogenized in extraction buffer (0.2 mM EDTA and 5mM Sodium Ascorbate in 50 mM potassium phosphate buffer, pH 7.0) using a chilled mortar and pestle. The homogenate was then centrifuged at 12 000xg for 20 min at 4°C. Supernatant was used for enzyme activity and protein content assays. All extracts were handled at 4°C. Total protein content of the enzyme extracts was determined following Bradford [35] using bovine serum albumin as a standard. All enzyme activities, except for Polyphenol oxidase, were expressed as U mg^-1^ of protein per minute.

Peroxidase (EC 1.11.1.7) activity was determined according to Fielding and Hall [36] by the oxidation of guaiacol in the presence of H_2_O_2_ using enzyme reaction solution containing 25 mM phosphate buffer (pH 7.0), 9 mM guaiacol, 10 mM H_2_O_2_ and enzyme extract. The increase of absorbance due to formation of tetraguaiacol was assessed at 470 nm [37]. The activity of ascorbate peroxidase (EC 1.11.1.11) was assayed using the method of Chen and Asada [38], by measuring the decrease in absorbance at 290 nm caused by ascorbic acid oxidation. In this assay, 50 mM phosphate buffer (pH 7), 0.1 mM EDTA, 5 mM H_2_O_2_ and 0.5 mM sodium ascorbate were used in the reaction solution. Catalase (EC 1.11.1.6) activity was assayed according to Aebi [39]. The decrease in absorbance at 240 nm was measured as a consequence of H_2_O_2_ consumption [40]. In this assay, 25 mM phosphate buffer (pH 7.8) and 30 mM H_2_O_2_ were used in the reaction solution. Polyphenol oxidase (EC 1.14.18.1) activity was determined according to the protocol described by [41]. The reaction mixture contained 0.1 mL of the sample solution and 0.5 ml catechol solution (1.6% in 70 mM phosphate buffer, pH 6.0). A unit of enzyme activity was defined as the change of 0.05 in the absorbance value under the conditions of the assay.

### Statistical analysis

The statistical analysis was performed using SPSS Ver.10 software (IBM Corporation and Others, Armonk, NY, USA) and a Two-way analysis of variance (ANOVA II). The data were expressed as the mean ± standard error. Means were statistically compared using Student-Newman-Keuls’s multiple-range test at the level of p < 0.05.

## Results

### Growth parameters analyses in *Vicia faba* genotypes grown under water deficit

**Fig 1:**
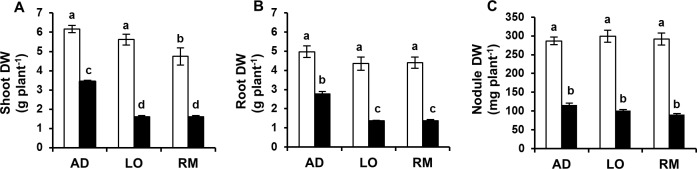
Growth performance responses to water deficiency. Twenty day-old *Vicia faba* plants, previously inoculated with the Fb41 rhizobia strain, were grown in control (white bars) or in water limited (black bars) conditions. A shoot, B root and C nodules dry weights (DW) were measured at plant flowering stage. AD: Aguadulce; LO: Luz d’Otono; RM: Reina Mora. The values shown are the mean ± SEM of three independent replicates. Differences in the data were considered significantly different at 0.05 level of probability by Student-Newman-Keuls Test (indicated by different letters).

Growth performances of three different faba bean genotypes were analysed under water deficit. The most common symptom of dehydration injury is the inhibition of growth, which is reflected in a reduction in the dry matter yield [42,43]. The root, shoot and nodule dry weight (DW) were analysed under well-watered and water-limited growth conditions (Fig 1). The results showed that water deficit significantly reduced growth performances. The root and nodule DW were similar for the three genotypes under control conditions. In contrast, the shoot DW of Reina Mora (“RM”) was significantly lower than Aguadulce (“AD”) and Luz d’Otonio (“LO”). Under water deficit, shoot, root, and nodule DW were significantly reduced compared to those achieved under control conditions. However, the shoot and root DW were significantly less reduced in “AD” than in “LO” and “RM” showing that “AD” plant growth is less affected by water limitation than “LO” and “RM”.

**Fig 2:**
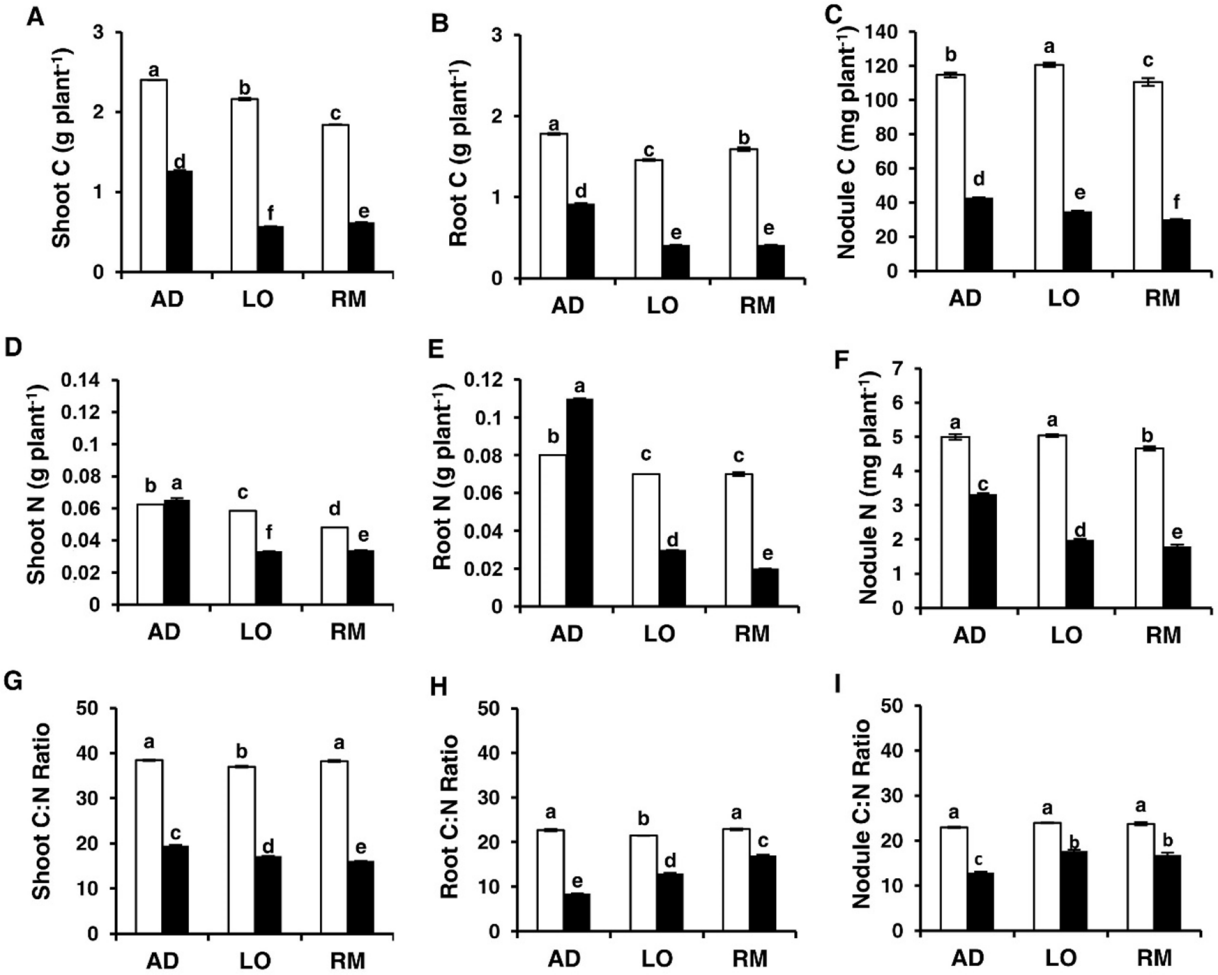
Changes in C/N balance parameters under water deficiency. Twenty day-old *Vicia faba* plants, previously inoculated with the Fb41 rhizobia strain, were grown in control (white bars) or in water limited (black bars) conditions. Carbon (A, B, C) and Nitrogen (D, E, F) contents were measured in shoots (A, D), roots (B, E) and nodules (C, F). Shoot (G), root (H) and nodule (I) C:N ratios were determined at plant flowering stage. AD: Aguadulce; LO: Luz d’Otono; RM: Reina Mora. The values shown are the means ± SEM of three independent replicates. Differences in the data were considered significantly different at the 0.05 level of probability by Student-Newman-Keuls Test (indicated by different letters).

The carbon-to-nitrogen ratio (C:N ratio) is an important parameter associated with optimal growth and plant development [44,45,46]. We analysed the C and N contents in roots, shoots and nodules of the three genotypes under well-watered and water-limited growth conditions (Fig 2). Amongst the different genotypes under control conditions, limited variations in C and N content of less than 25% for C content and 15% for N content were observed for the different organs tested. Under water deficit, the C content of shoots and roots was more reduced in “LO” and “RM” than in “AD”. The N content also decreased significantly in “LO” and “RM” organs under water deficit. In contrast, for the “AD” genotype, we noticed an increase of N content in shoots (17%) and roots (38%) of stressed plants. Analysis of the C:N ratio in the different organs showed a significant decrease under water deficit. The reduction of the C:N ratio in roots (64%) and nodules (44%) of “AD” was significantly more important than in roots and nodules of “LO “and “RM”. For shoots, the reduction of the C:N ratio was around 50% in “AD”, “LO” and “RM”.

Taken together, these results showed that the C:N ratios were significantly reduced in all the considered organs. However, the increased N content in roots and shoots of “AD” under water deficit is a striking difference with “LO” and “RM” genotypes.

### Physiological and photosynthetic responses to water deficit in leaves

**Fig 3:**
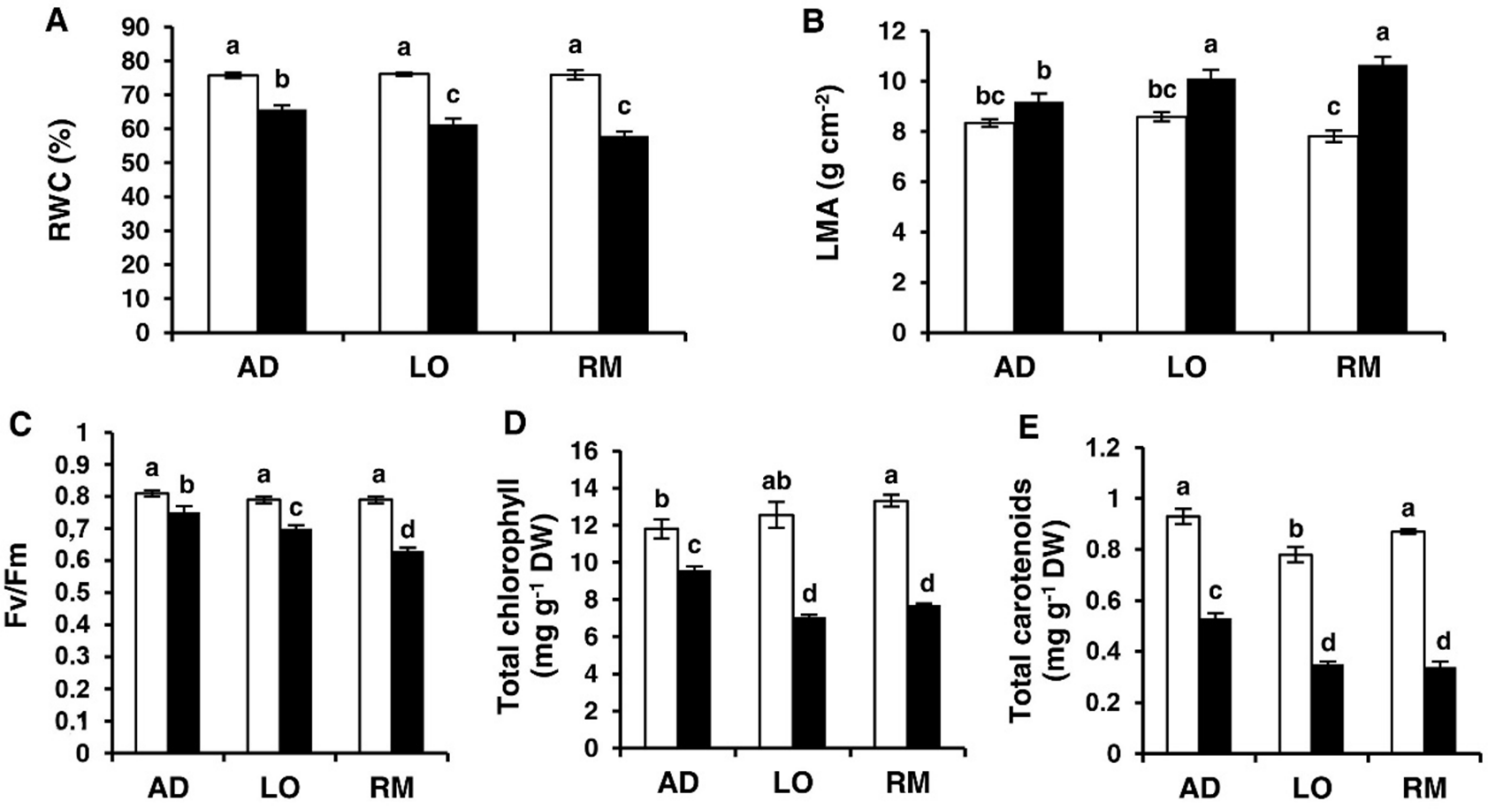
Water and leaf parameter responses to water deficiency. Twenty day-old *Vicia faba* plants, previously inoculated with the Fb41 rhizobia strain, were grown in control (white bars) or in water limited (black bars) conditions. At plant flowering stage, fully expanded third leaves were used for the measurements. (A) Leaf relative water content (RWC), (B) leaf mass area (LMA), (C) efficiency of photosystem II (Fv/Fm), (D) Total chlorophyll, (E) Carotenoids contents. AD: Aguadulce; LO: Luz d’Otono; RM: Reina Mora. The values shown are the means ± SEM of three independent replicates. Differences in the data were considered significantly different at the 0.05 level of probability by a Student-Newman-Keuls Test (indicated by different letters).

The effect of water deficit on faba bean was analysed based on multiple leaf physiological markers (Fig 3). Relative water content (RWC) is widely used to describe the water balance of plants and the leaf mass area (LMA) is a good morphological trait of plant functioning. Chlorophyll fluorescence (Fv/Fm) is commonly used as an index of photosystem functioning [47]. Finally, the chlorophyll (Chl) and carotenoid (Car) contents were used as biochemical markers, since leaf Car content and their proportion to Chl are widely used for diagnosing the physiological state of plants during acclimation and adaptation to different stresses. RWC, LMA, and Fv/Fm did not vary significantly between the different cultivars under control conditions. RWC was significantly reduced in the three genotypes under water limited conditions and this reduction was significantly lower in “AD” (13%) than in “LO” (19%) and “RM” (24%). Similarly, water deficit significantly increased LMA in “AD” (10%) but this increase was lower than in “LO” (18%) and in “RM” (36%). Photosynthetic parameters (Fv/Fm and Chl/Car contents) were significantly reduced under water limitation conditions. However, as for RWC and LMA, the reduction of these parameters was less important in “AD” than in “LO” and “RM”. Altogether, the results showed that the lower alterations in measured parameters observed in “AD” are correlated with the lower reduction in shoot, root and nodule dry weight suggesting that the lower impairments of physiological parameters allowed better plant growth conditions under water limited conditions.

### Accumulation of osmolytes in response to water deficit

**Fig 4:**
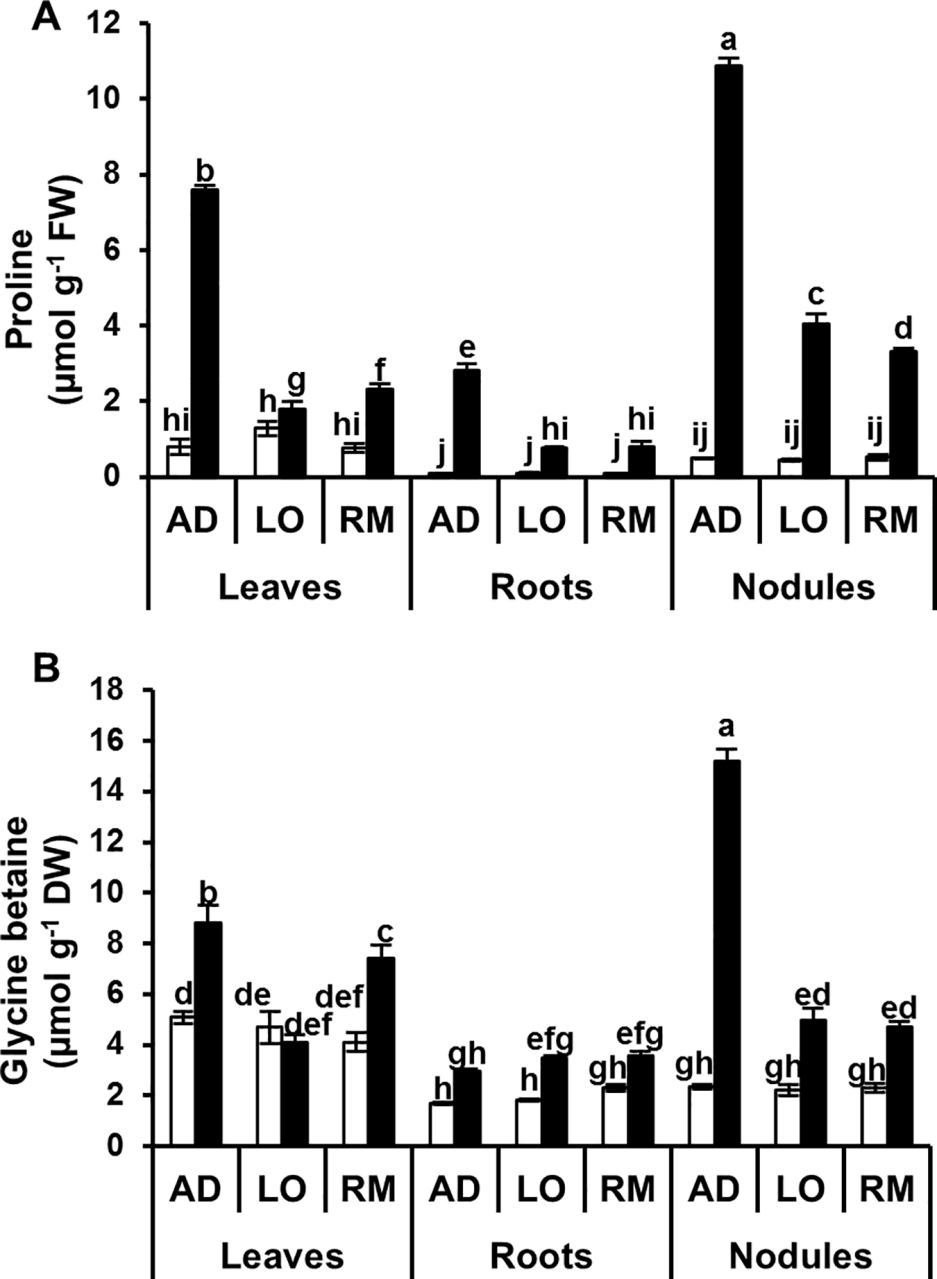
Proline and glycine betaine contents, in leaves, roots and nodules. Twenty day-old *Vicia faba* plants, previously inoculated with the Fb41 rhizobia strain, were grown in control (white bars) or in water limited (black bars) conditions. At plant flowering stage, samples from fully expanded third leaves, roots and nodules were used for the measurements of proline (A) and glycine betaine (B) contents. Pro: proline; GB: glycine betaine; AD: Aguadulce; LO: Luz d’Otono; RM: Reina Mora. The values shown are the means ± SEM of three independent replicates. Differences in the data were considered significantly different at the 0.05 level of probability by a Student-Newman-Keuls Test (indicated by different letters).

Proline and glycine betaine (GB) are known osmolytes produced by plants under drought stress. We measured the content of these two molecules in leaves, roots and nodules (Fig 4). Proline contents were not significantly different in the organs of the three cultivars under control conditions. Under hydric stress, proline content strongly increased in all organs of the different cultivars. The highest accumulations were observed in roots and nodules under water limitation with a hundred to a thousand fold induced proline accumulation compared to controls. The highest accumulations were observed in “AD” with 800%, 3000% and 2100% increases in leaves, roots and nodules, respectively. The increase in proline accumulation was significantly higher in “AD” than in the two other genotypes. GB contents were not significantly different in the organs of the three cultivars under control conditions. GB accumulated in the different organs of the three genotypes under water-limited conditions except in leaves of “LO”. The most striking difference between the three cultivars was the strong GB accumulation in nodules of “AD”. This strong GB accumulation can be associated to both the plant and the rhizobia in which GB also provides an enhanced level of osmotic stress tolerance [48]. Overall, the significant GB accumulation in “AD” nodules could be related to a better resilience of the nitrogen-fixing interaction to water deficit. In contrast, no clear trend was observed in GB accumulation in roots and leaves of the three cultivars under water limitation.

### Redox modifications in response to water deficit

**Fig 5:**
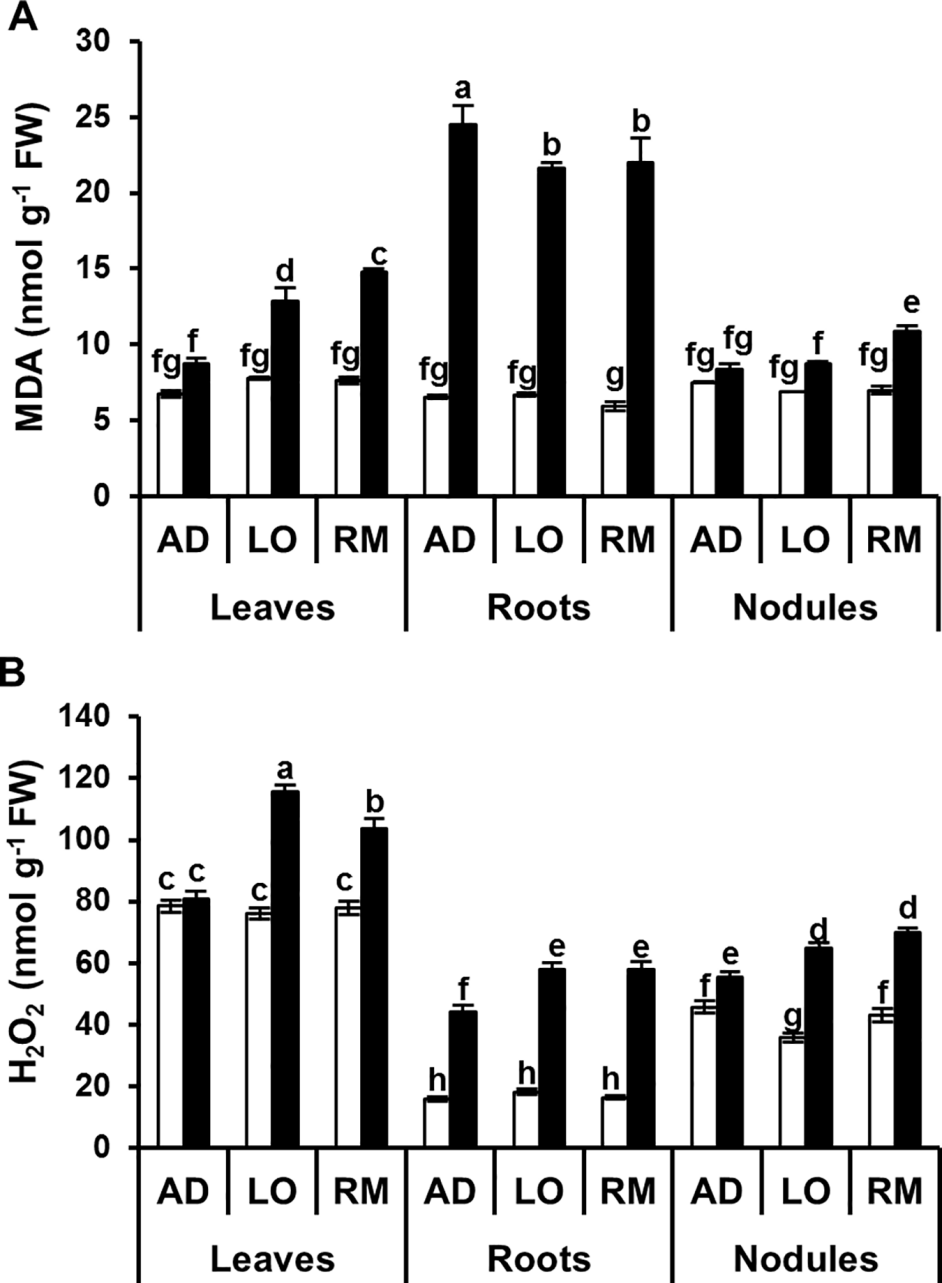
Malondialdehyde and hydrogen peroxide contents in leaves, roots and nodules. Twenty day-old *Vicia faba* plants, previously inoculated with the Fb41 rhizobia strain, were grown in control (white bars) or in water limited (black bars) conditions. At plant flowering stage, samples from fully expanded third leaves, roots and nodules were harvested and used for the measurements of malondialdehyde (A) and hydrogen peroxide (B) contents. MDA: malondialdehyde; H_2_O_2_: hydrogen peroxide. AD: Aguadulce; LO: Luz d’Otono; RM: Reina Mora. The values shown are the means ± SEM of three independent replicates. Differences in the data were considered significantly different at the 0.05 level of probability by a Student-Newman-Keuls Test (indicated by different letters).

Under stress, cellular homeostasis disruption induces lipid peroxidation with the accumulation of malondialdehyde (MDA) and ROS production such as hydrogen peroxide (H_2_O_2_). We measured the content of MDA and H_2_O_2_ in leaves, roots and nodules (Fig 5). MDA contents were significantly increased in the considered organs of all tested genotypes under water-limited conditions. No significant difference was observed in MDA accumulation in roots and nodules of the three cultivars. In contrast, MDA accumulation was significantly lower in leaves of “AD” than in the two other cultivars, “LO” and “RM”. Under control conditions, H_2_O_2_ content was similar in leaves and roots of the three genotypes. Under water deficit, H_2_O_2_ content increased in all the organs of the three genotypes compared to controls. However, this increase was always significantly lower in “AD” than in “LO”. Moreover, the H_2_O_2_ content in “AD” leaves under limiting water conditions was not significantly different compared to the control conditions. Taken together, the results showed that better growth performance of the “AD” genotype under water limitation is correlated to the lower accumulation of H_2_O_2_ in the different organs and of MDA in leaves and nodules compared to “LO” and “RM” genotypes.

**Fig 6:**
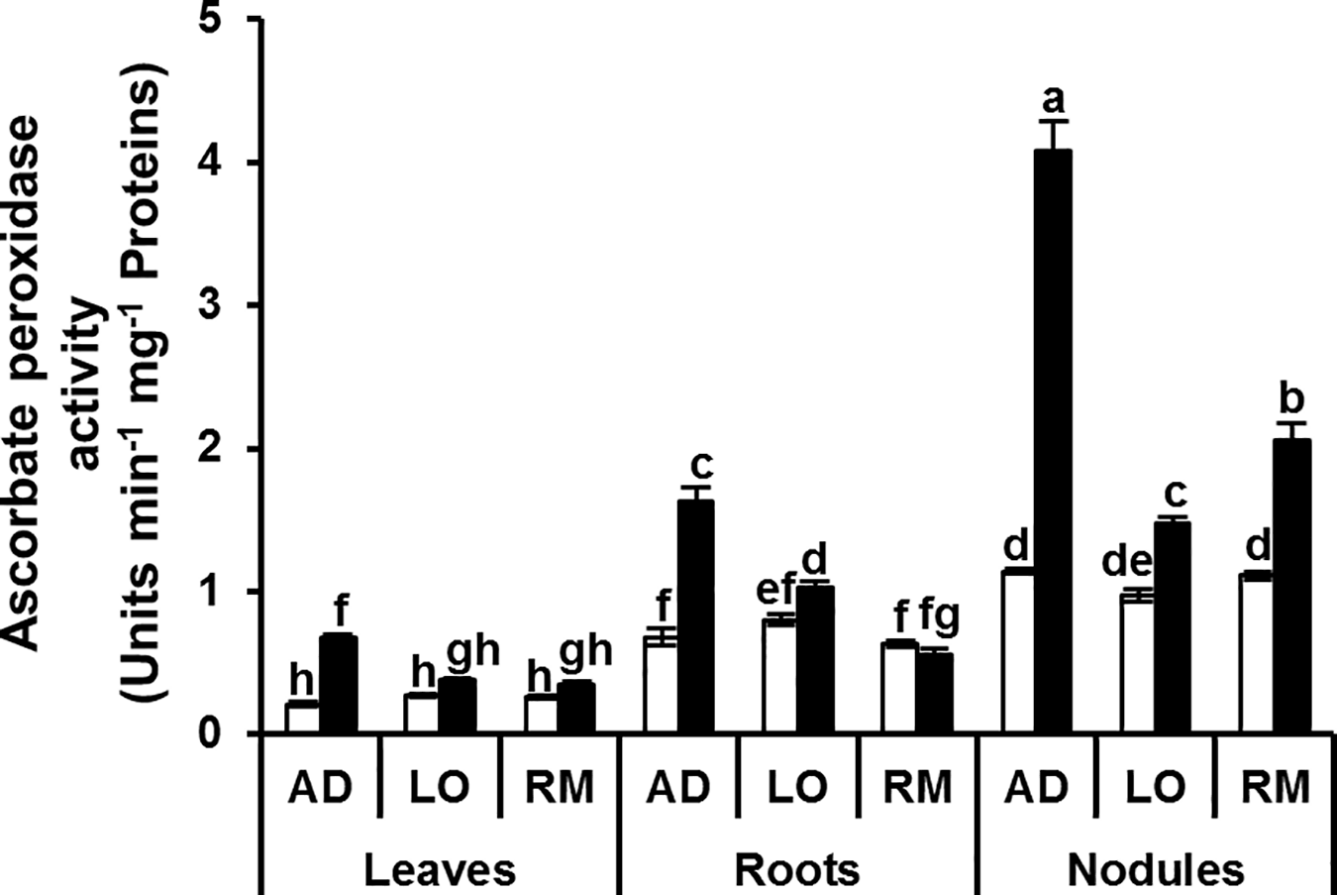
Ascorbate peroxidase activity in leaves, roots and nodules. Twenty day-old *Vicia faba* plants, previously inoculated with the Fb41 rhizobia strain, were grown in control (white bars) or in water limited (black bars) conditions. At plant flowering stage, samples from fully expanded third leaves, roots and nodules were harvested and ascorbate peroxidase activity was assayed. AD: Aguadulce; LO: Luz d’Otono; RM: Reina Mora. The values shown are the means ± SEM of three independent replicates. Differences in the data were considered significantly different at the 0.05 level of probability by a Student-Newman-Keuls Test (indicated by different letters).

**Fig 7:**
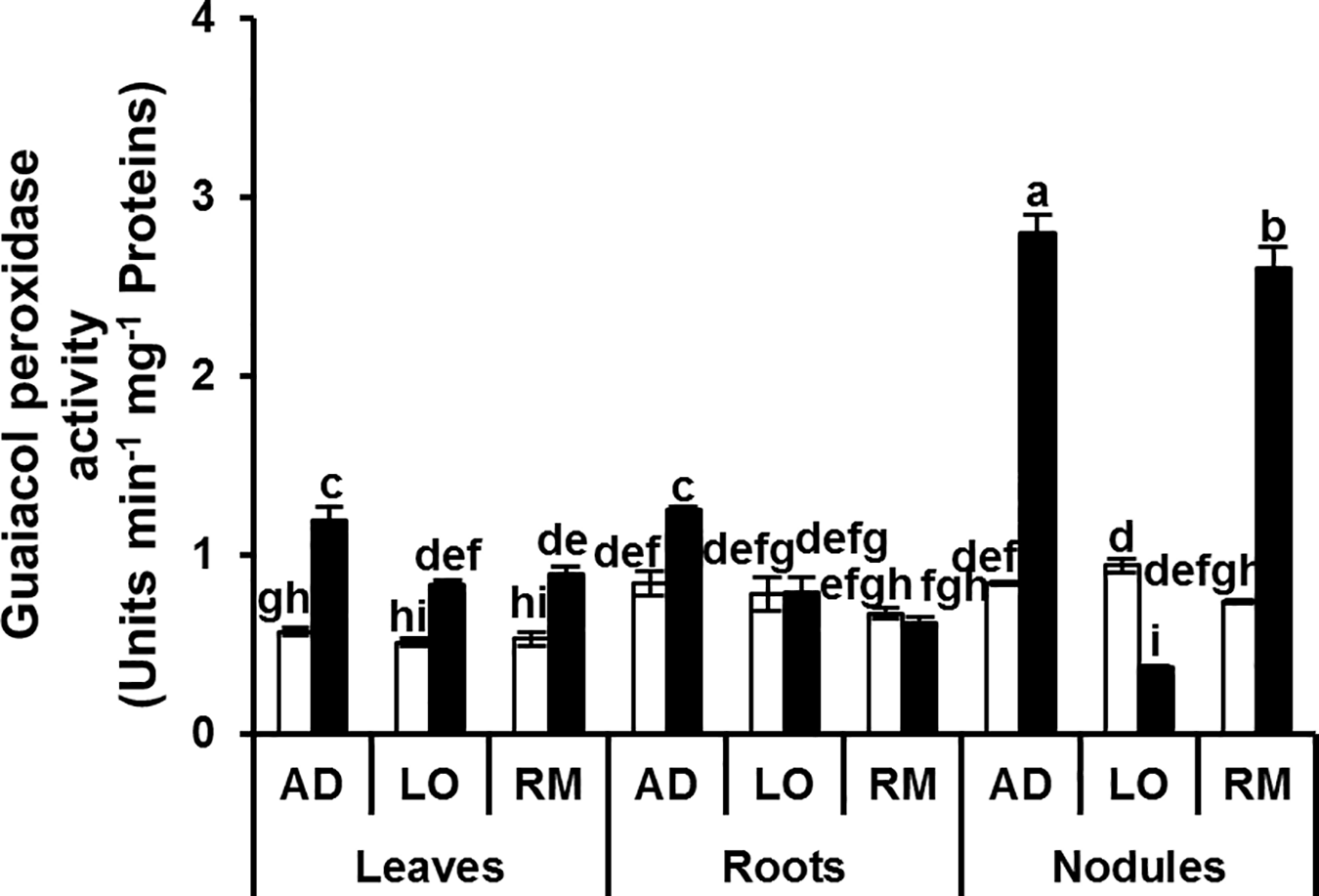
Guaiacol peroxidase activity in leaves, roots and nodules. Twenty day-old *Vicia faba* plants, previously inoculated with the Fb41 rhizobia strain, were grown in control (white bars) or in water limited (black bars) conditions. At plant flowering stage, samples from fully expanded third leaves, roots and nodules were harvested and guaiacol peroxidase activity was assayed. AD: Aguadulce; LO: Luz d’Otono; RM: Reina Mora. The values shown are the means ± SEM of three independent replicates. Differences in the data were considered significantly different at the 0.05 level of probability by a Student-Newman-Keuls Test (indicated by different letters).

To cope with this stress-induced oxidative imbalance, plant cells activate ROS scavenging and antioxidant systems. The activities of antioxidant enzymes ascorbate peroxidase (APX), guaiacol peroxidase (GaPX), catalases (CAT) and polyphenol oxidases (PPO) were measured as parameters of antioxidant defence. APX and GaPX activities were significantly higher in roots and nodules than in leaves of the three genotypes under control conditions (Figs 6 and 7). Both APX and GaPX activities were more induced in the different organs of “AD” than in “LO” and “RM”.

**Fig 8:**
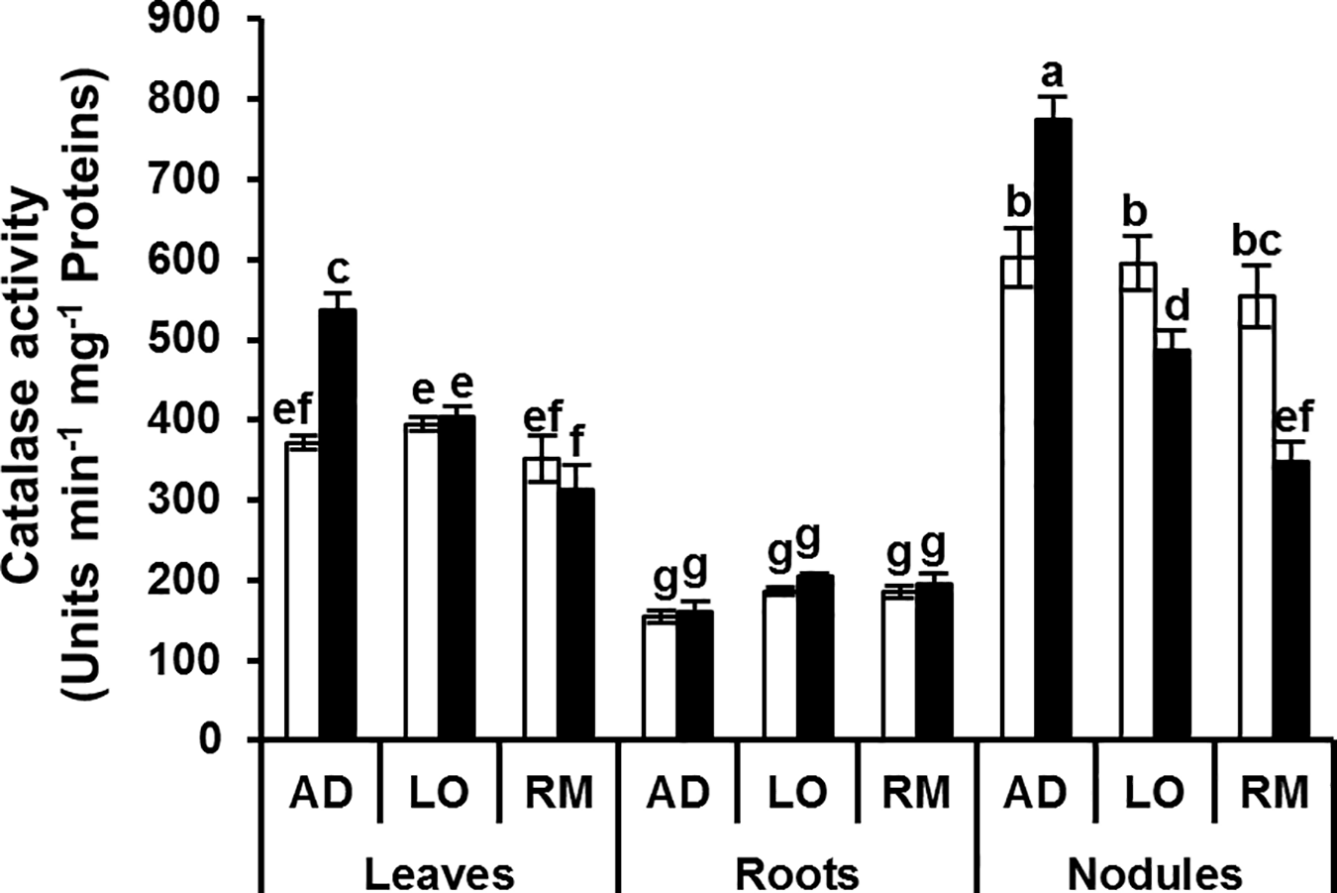
Catalase activity in leaves, roots and nodules. Twenty day-old *Vicia faba* plants, previously inoculated with the Fb41 rhizobia strain, were grown in control (white bars) or in water limited (black bars) condition. At plant flowering stage, samples from fully expanded third leaves, roots and nodules were harvested and catalase activity was assayed. AD: Aguadulce; LO: Luz d’Otono; RM: Reina Mora. The values shown are the means ± SEM of three independent replicates. Differences in the data were considered significantly different at the 0.05 level of probability by a Student-Newman-Keuls Test (indicated by different letters).

**Fig 9:**
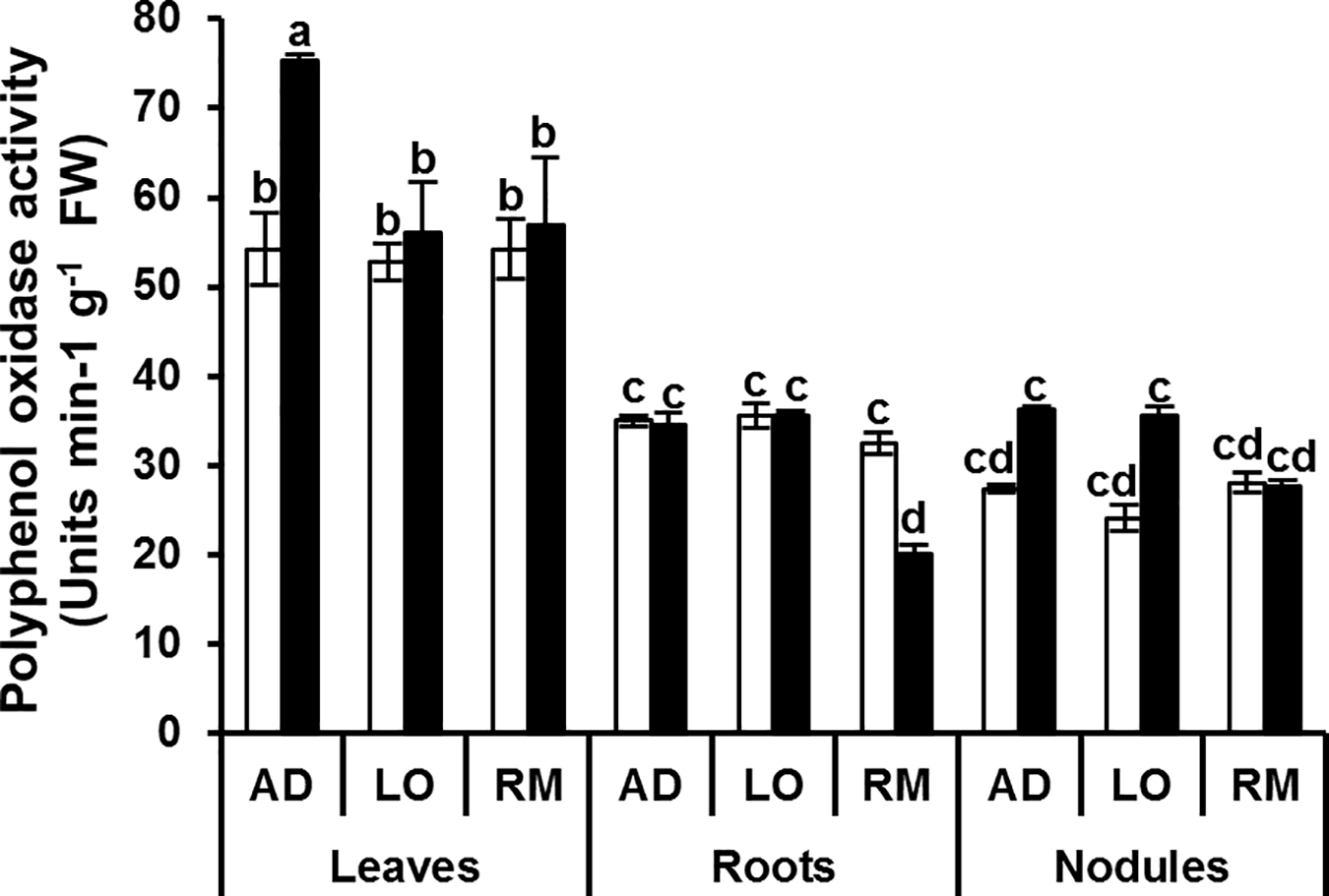
Polyphenol oxidase activity in leaves, roots and nodules. Twenty day-old *Vicia faba* plants, previously inoculated with the Fb41 rhizobia strain, were grown in control (white bars) or in water limited (black bars) conditions. At plant flowering stage, samples from fully expanded third leaves, roots and nodules were harvested and polyphenol oxidase activity was assayed. AD: Aguadulce; LO: Luz d’Otono; RM: Reina Mora. The values shown are the means ± SEM of three independent replicates. Differences in the data were considered significantly different at the 0.05 level of probability by a Student-Newman-Keuls Test (indicated by different letters).

In contrast to APX and GaPX activities, CAT and PPO activities did not show a clear general induction pattern associated to the better growth performance of “AD” (Figs 8 and 9). Nevertheless, both CAT and PPO activities were significantly up regulated in leaves of “AD” and not in leaves of “LO” and “RM”.

All results considered, the low increase of MDA and H_2_O_2_ content observed in leaves and nodules of “AD” compared to the two other genotypes is correlated to the increased antioxidant activities detected in the “AD” genotype.

## Discussion

**Fig 10:**
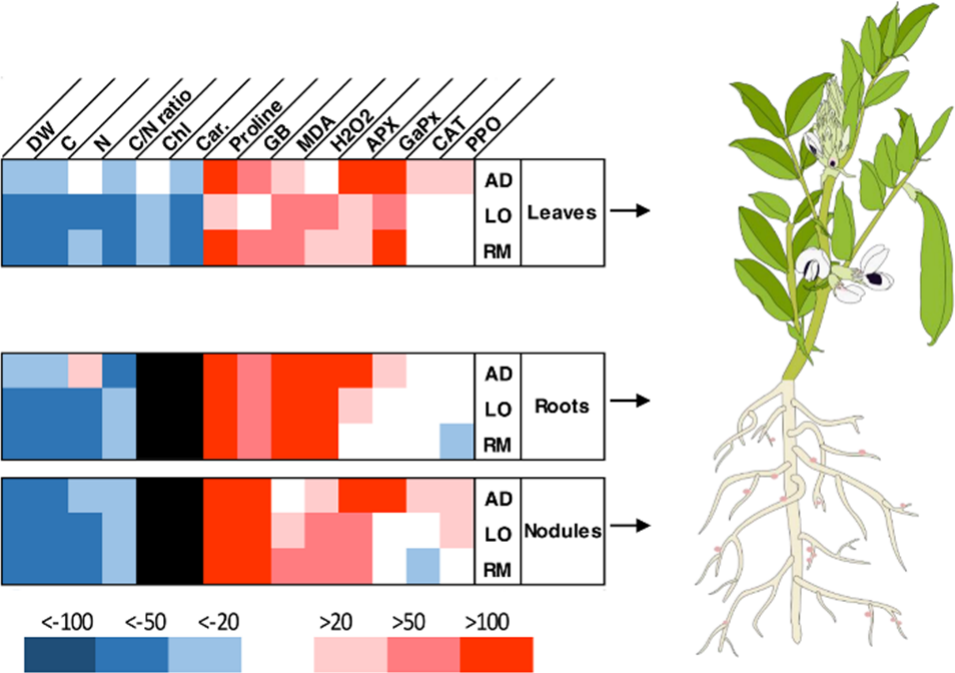
Summary of the *Vicia faba* responses to drought stress. The modification of the biological markers in *V*. *faba* plants under water-limited conditions is indicated in percentage of the level of the marker for plants in controlled conditions. DW: dry weight; C: carbon; N: nitrogen; Fv/Fm: maximal quantum yield of PSII photochemistry; RWC: relative water content; LMA: leaf; Chl: chlorophyll; Car.: carotenoids; GB: glycine betaine; MDA: malondialdehyde; APX: ascorbate peroxidase; GaPX: guaiacol peroxidase; CAT: catalase; PPO: polyphenol oxidase. AD, Aguadulce; LO, Luz de Otonio; RM, Reina Mora.

The level of plant tolerance or sensitivity to water stress depends on the species and genotype, length and severity of water loss, as well as on the developmental stage. Moreover, drought tolerance is the specific adaptation to water deficit through organs or processes that facilitate tolerance [49]. The goal of this study was to analyse the physiological and biochemical responses of three *V*. *faba* genotypes under mild water deficit conditions and to identify reliable biological markers correlating plant adaptation to water deficit. Our results showed that water deficit significantly decreased the plant growth of the three *V*. *faba* genotypes. However, “AD” presented a significantly higher leaf and root DW than “LO” and “RM”, showing that amongst the most common genotypes used in Morocco, “AD” is the one that presents the better growth under water deficit conditions (Fig 10). In contrast to leaf and root DW, the DW of “AD” nodules was affected in a similar way to that of “LO” and “RM” showing a similar impact of water stress on nodule growth. Similarly, Coleto et al. [50] reported that drought affected nodules more severely than other plant tissues in common bean.

Numerous physiological and biochemical parameters were tested in the three genotypes to identify robust markers associated to the better plant growth of “AD” in limiting water conditions. The reduction of C content is significantly lower in all the organs of “AD” than in those of “LO” and “RM”. However, the use of this parameter may be difficult as the downward trend is similar in the three genotypes. In contrast, root and shoot N content was higher in “AD” whereas it is reduced in the same organs of “LO” and “RM” under water deficit suggesting that the biological nitrogen fixation and nitrogen transport are significantly more efficient in “AD” than in “LO” and “RM”. Thus, shoot and root N contents appear to be good markers to screen for a better *V*. *faba* adaptation to water deficit (Fig 10). Analysis of the C:N ratio shows different results depending on the organs analysed and does not seems to be easily applicable to differentiate resistant and sensitive genotypes. These results are linked to a general higher C and N contents in “AD” than in “LO” and “RM” under water deficit. This suggests that, as for N nutrition, C assimilation via photosynthesis and transport are more effective in AD than in “LO” and “RM” under water deficit.

Analysis of leaf parameters (RWC, LMA, Fv/Fm, total chlorophyll and carotenoid content) showed that the “AD” genotype presented the lowest alteration of these five parameters compared to “LO” and “RM”. Thus, these results support the idea that “AD” is less affected than “LO” and “RM” under water deficit. Amongst these markers, LMA which is not significantly different in control and stressed “AD”, and total chlorophyll which is not highly reduced under water deficit in “AD” compared to “LO” and “RM” appear to be two robust markers of *V*. *faba* adaptation to water deficit.

Proline and glycine betaine (GB) are potent osmoprotectants accumulated under water deficit [51]. Proline was accumulated in all the different organs of the three genotypes. However, this accumulation was significantly higher in “AD” than in the two others genotypes. In contrast, GB does not seem to be a trait associated with tolerance in faba bean since “LO” and “RM” accumulated more GB in root and shoot than “AD”, respectively. In many plants, proline accumulation has been found to be correlated with drought-stress tolerance, although in some cases no correlation could be found (reviewed in Szabados and Savouré [52]). In *V*. *faba*, the four-fold higher accumulation of proline in “AD” compared to the other genotypes indicate that proline content is a good marker for plant resistance to water deficit. A similar conclusion was suggested from a recent study in field experiments [53].

Analysis of the different organs showed accumulation of MDA and H_2_O_2_ under water deficit. However, significant lower accumulation of H_2_O_2_ in all the organs and of MDA in shoots was detected in “AD” compared to “LO” and “RM”. These results suggest that oxidative stress is less marked in “AD” than in the two other genotypes. Interestingly, H_2_O_2_ accumulation was similar in leaves of “AD” in control and water limited conditions (Fig 5). In the search for good selection markers, H_2_O_2_ accumulation in leaves should allow resistant genotypes to be differentiated from sensitive ones.

The lower level of H_2_O_2_ correlated with the better water deficit adaptation suggests that detoxification mechanisms could be more efficient in the “AD” genotype than in the two other genotypes. The induction of the cellular antioxidant machinery plays a crucial role for protection against drought-induced oxidative stress. In our experiments, APX activity increased significantly in leaves, roots and nodules of “AD” under water deficit. This increase was not observed in the two other genotypes which grew less well under water deficiency (Fig 10). In a recent study, Abid and colleagues similarly showed enhanced leaf antioxidant activities (CAT, SOD, GaPX) in two faba bean cultivars (‘Giza 3’ and ‘Hara’) although no significant difference was observed in APX activity [54]. This may highlight the great drought tolerance of AD cultivar obtained in our experiments. The results for GaPX activity were also clear in roots where this activity was significantly induced in “RM” but not in the two other genotypes. For catalase activity, significant induction was detected in leaves and nodules of “AD” whereas there was no significant induction in “LO” and “RM”. A significant reduction in catalase activity was even detected in the “LO” and “RM” nodules. Finally, a significant induction in polyphenol oxidase activity was found in the leaves of “AD” whereas no difference was detected in leaves of “LO” and “RM” showing that this activity is potentially a good marker for water deficit tolerance in *V*. *faba* (Fig 10). The better growth performance of the “AD” genotype is correlated to its ability to induce its antioxidant enzymatic defence. This may contribute to protect the general metabolic functioning of the plant. The higher C and N contents observed in “AD” strongly suggest that both photosynthesis and biological nitrogen fixation are more efficient in “AD” than in “LO” and “RM” under water deficit. These data are in agreement with other studies reporting the increased activity of antioxidant enzymes in faba bean in response to drought stress [55,54,56]. These authors found higher values for these enzymatic activities in tolerant genotypes such as ‘Hara’ ‘C5’ and ‘Zafar 1’ than in sensitive genotypes [55,56].

Nitrogen content was more important in “AD” than in “LO” and “RM” suggesting that the biological nitrogen fixation is more efficient in “AD” than in “LO” and “RM”. Analyses of osmolyte accumulation showed that both proline and glycine betaine accumulate more in AD nodules than in “LO” and “RM” nodules. Increased osmolyte accumulation is correlated to an enhanced tolerance to drought stress [57,58] and better nitrogen-fixing activity in transgenic *Medicago truncatula* plants [57]. In our work, the four-fold higher accumulation of proline in “AD” root nodules under water stress compared to the other genotypes, shows that proline content is strongly increased compared to “LO” and “RM” even if all tested genotypes showed increased of proline content. These results are consistent with previously reported data for faba bean, where drought tolerant genotypes accumulate more proline than sensitive genotypes [55,56], even if nodule proline content was not evaluated. A similar conclusion was suggested from a recent study in field experiments [53]. In addition to proline, glycine betaine content was also ten-fold higher in “AD” root nodules compared to “LO” and “RM”. This accumulation was significantly higher than in roots and leaves of the same genotype. It may be hypothesized that the glycine betaine synthesis may be used as carbon and/or nitrogen sources by the bacterial partner [59]

A common feature of nodule drought stress is a decreased catalase activity in nodule [60,61,62,63]. CAT is not regarded as the forefront of antioxidant defence under osmotic stress because of the low affinity to its substrate [64,65], although this enzyme plays an important role in the nodulation process and nodule functioning [66,67]. In our work, we showed a significantly increased catalase activity in AD root nodules compared to LO and RM. Interestingly, this higher activity was associated with a decreased H_2_O_2_ content. The higher catalase activity in root nodules may be associated to both symbiotic partners and it may play a significant role in nitrogen fixation efficiency [68]. Thus, nodule catalase activity may be used as a biological marker to screen *V*. *faba* genotypes tolerant to mild water stress conditions.

## Conclusions

Based on this research, we suggest that the “AD” genotype presents a higher tolerance to water deficiency than “LO” and “RM”. Thereby, the “AD” genotype may be considered as an interesting candidate to be used in crosses for tolerance breeding. In parallel, our study shows that various parameters are differentially altered in the genotypes depending on their tolerance levels. Both “LO” and “RM”, two genotypes with a lower resistance to water deficit, show a similar behavior which is different from that of “AD”. The different biological markers that show the most differentiated profiles will be helpful to screen for water deficit tolerant *V*. *faba* genotypes in future breeding programs.

## Abbreviations

AD: Aguadulce
ANOVA: analysis of variance
APX: ascorbate peroxidase
CAT: catalase
Car: carotenoids
Chl: chlorophyll
DW: dry weight
FC: field capacity
Fv/Fm: maximal quantum yield of PSII photochemistry
FW: fresh weight
GaPX: guaiacol peroxidase
GB: glycine betaine
LMA: leaf mass area
LO: Luz d’Otonio
MDA: malondialdehyde
PPO: polyphenol oxidase
Pro: proline
RM: Reina Mora
RWC: relative water content
